# 胸腔镜在肺部微小结节诊治中的应用

**DOI:** 10.3779/j.issn.1009-3419.2013.07.07

**Published:** 2013-07-20

**Authors:** 立群 单, 坚 胡, 明东 李, 翀 张, 夏轶 闾

**Affiliations:** 1 310003 杭州，浙江大学医学院附属第一医院胸外科 Department of Thoracic Surgery, the First Affiliated Hospital of Medical School of Zhejiang University, Hangzhou 310003, China; 2 317500 温岭，温岭市第一人民医院胸外科 Department of Thoracic Surgery, the First People's Hospital of Wenling City, Wenling 317500, China

**Keywords:** 胸腔镜, 肺微小结节, CT引导的hook-wire定位, Video-assisted thoracoscopic surgery, Small pulmonary nodules, CT-guided hook-wire localization

## Abstract

**背景与目的:**

随着胸部CT的应用推广，孤立性肺结节的出现逐渐增多，尤其是胸部薄层高分辨CT的检查，检出直径10 mm以下的微小结节的概率增高，临床上对于如何诊治这类微小结节仍存在较大争议。本研究旨在探讨胸腔镜手术在肺部微小结节的诊断和治疗中的价值。

**方法:**

2009年11月-2012年5月，对64例肺微结节应用电视胸腔镜手术先行肺结节楔形切除，术前均无明确病理诊断，术中送快速冰冻病理检查，根据病理结果 < 综合考虑后决定手术方式。

**结果:**

原发性肺癌行全胸腔镜肺叶切除+淋巴结清扫20例; 行肺楔形切除44例，其中良性肿瘤21例、癌前病变18例、转移癌3例、不适合肺叶切除的原发性肺癌2例。所有患者均明确病理诊断，确诊率100%。全组患者手术顺利，无严重手术并发症和围手术期死亡。

**结论:**

肺部微小结节影像学诊断有一定难度; 胸腔镜手术诊治肺部微小结节创伤小、诊断准确，具有重要的诊断意义。术前CT引导的hook-wire定位可帮助手术中探查病变部位。

## Abstract (en)

**Background and objective:**

Chest computed tomography (CT), particularly thin-slice high resolution CT, has low sensitivity and specificity for detecting pulmonary nodules < 10 mm in size. This limitation leads to challenges in clinical diagnosis and treatment of small pulmonary nodules. This study introduces the use of video-assisted thoracoscopic surgery (VATS) for the diagnosis and treatment of small pulmonary nodules.

**Methods:**

From November 2009 to May 2012, 64 patients with small pulmonary nodules without prior preoperative pathologic diagnosis were treated by pulmonary wedge resection through VATS. The diagnosis of small pulmonary nodules was established from rapid frozen section. The type of operation depends on the pathology and the condition of the patients. Twenty patients with primary lung cancer were subjected to lobectomy and radical resection of the lymph nodes by complete thoracospic lobectomy or video-assisted thoracoscopic invasive lobectomy. Pulmonary wedge resection was performed in 44 patients, among whom 21 have benign nodule, 18 have precancerous lesion, 3 have metastatic nodule, and 2 have primary lung cancer for which lobectomy was not fit.

**Results:**

Confirmative diagnosis is difficult to obtain among patients with small pulmonary nodules. VATS is effective in the diagnosis and treatment of small pulmonary nodules. With VATS, patients with benign small pulmonary nodules can be cured, and patients with primary lung cancer can receive definite diagnosis and effective treatment in time.

**Conclusion:**

CT-guided hook-wire fixation is useful in precise lesion localization for surgical resection.

孤立性肺结节是指肺实质内的圆形或椭圆形致密影，通常直径≤3 cm，多数不伴有纵隔淋巴结肿大、肺炎、肺不张、胸腔积液等其它病变^[1]^，而直径≤1 cm者则称为微小结节。随着计算机断层扫描（computed tomography, CT）等影像技术的广泛应用，临床上肺微小结节的出现逐渐增多，但如何及时明确诊断是一大难题。传统X线、CT、磁共振成像（magnetic resonance imaging, MRI），甚至是正电子发射计算机断层显像（positron emission tomography computed tomography, PET-CT）等检查均难以明确肺微小结节的诊断; CT引导下的肺穿刺活检及纤维支气管镜检查的阳性率也不令人满意^[2]^，采取定期影像学随访观察结节变化，可能延误治疗，使部分恶性肿瘤进展，也会对部分患者产生巨大的心理压力。本研究回顾性分析了2009年11月-2012年5月在我中心通过胸腔镜诊治的64例肺部无症状孤立性微小结节，以探讨胸腔镜在肺孤立性微小结节诊治中的应用的意义，现报道如下。

## 1 资料与方法

### 1.1 一般资料

本组64例，男39例，女25例。年龄14岁-83岁，平均53.5岁，其中14岁1例，22岁-60岁33例，61岁-83岁30例。咳嗽5例，胸痛4例，胸闷1例，无任何症状体检发现54例。查体均无明显异常。病例选择标准：肺周围≤1 cm的圆形或类圆形结节，其中直径 < 5 mm的13例，5 mm≤直径≤10 mm的51例，均无阻塞性肺炎、肺不张、胸腔积液、纵隔淋巴结肿大等其它病变，术前均行纤维支气管镜检查，其纤维支气管镜检查及痰细胞学检查均为阴性，术前均无病理诊断。

### 1.2 方法

对于直径 < 1 cm，距脏层胸膜深度 > 0.5 cm的小结节，术中较难触及，宜采用CT引导下肺小结节Hook-wire定位^[3]^。本文中35例患者因此采用Hook-wire定位，而另29例患者因结节位于肺表面，且CT示其密度较高，考虑根据解剖位置及探查可触及肿块，故未予定位。双腔气管插管全身麻醉，健侧卧位健侧肺通气。分别于患侧腋中线第7肋间做一1.5 cm切口、腋前线第4肋间做一2 cm-3 cm切口及腋后线第9肋间做一个2 cm切口，于腋中线切口置入trocar，置入胸腔镜。对于已行Hook-wire定位的患者，根据定位部分再次探查结节，探查到结节后予切割缝合器距肿块边缘2.0 cm以上楔形切除肿块（图 1，图 2）;

**1:**
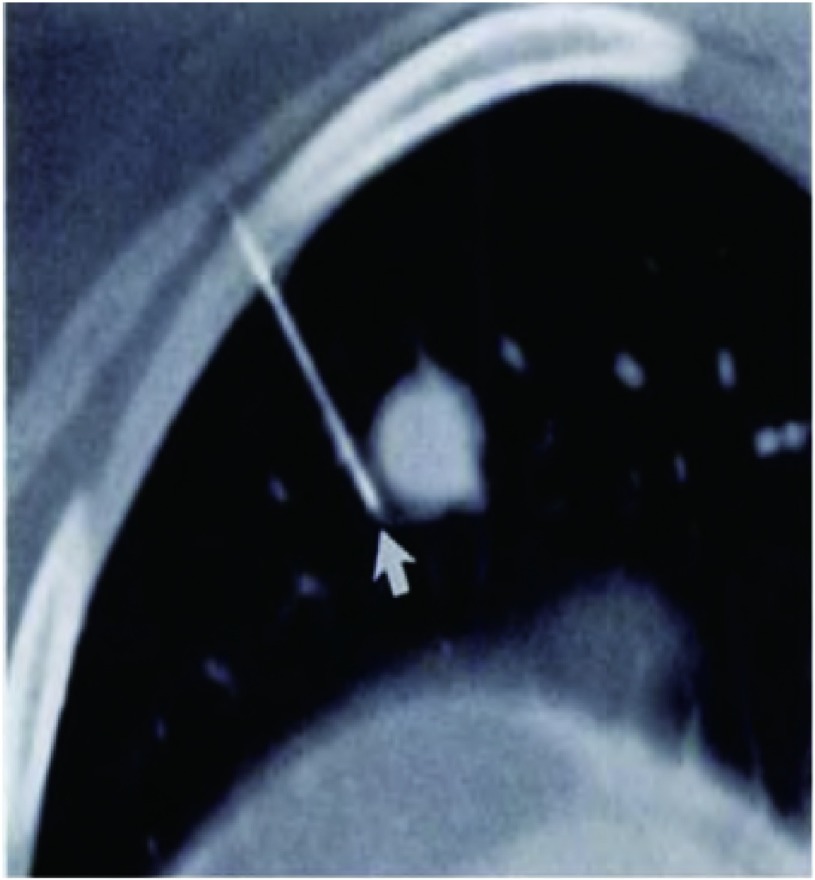
Hook-wire钢丝穿过结节旁 Hook-wire through the nodule ege

**2:**
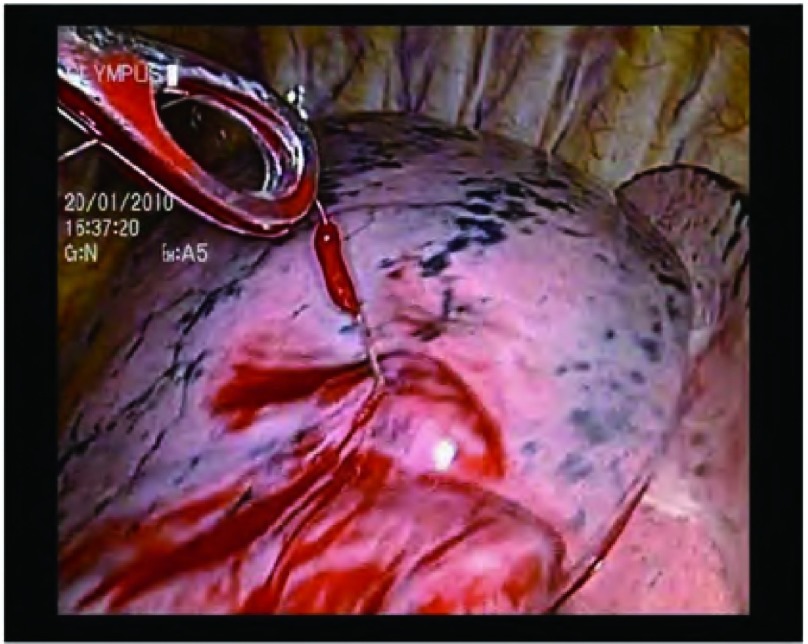
根据hook-wire定位探查结节 Exploration of the nodule by the hook-wire localization

对于未定位的患者，在胸腔镜引导下采用卵圆钳或手指探查，探查到肺内微小结节后，根据病变的情况置入操作器械，用抓钳提起肿物，距肿物边缘2.0 cm以上用切割缝合器完整切除肿物结节，取出送快速冰冻病理检查; 若冰冻病理检查结果是良性、癌前病变或是转移瘤，则置入胸管排除出血和漏气等情况和关胸结束手术，若病理证实为原发性肺癌，则在胸腔镜下行肺叶切除+淋巴结清扫手术。

## 2 结果

本组64例术中快速冰冻病理诊断为恶性病变25例，其中20例行全胸腔镜肺叶切除及系统性淋巴结清扫术，3例转移癌行病灶局部切除，2例原发性肺癌因肺功能差单纯行楔形切除。21例术中快速病理诊断为良性病变，行病灶局部切除。18例术中快速病理诊断为癌前病变，予其大楔形切除，1例行完全胸腔镜肺叶切除术中因肺动脉分支出血，镜下予缝合止血并完成手术，余手术均顺利。术中出血量10 mL-500 mL，平均（150±50）mL。术后发生胸腔积液1例，肺部感染1例，胸管拔除超过1周者1例，均经相应处理后好转，无严重术后并发症及围手术期死亡。本组64例术后均获得明确病理诊断，恶性肿瘤25例（39.1%）：腺癌13例，肺泡细胞癌4例，鳞癌5例，不典型类癌1例，转移性癌2例。良性病变21例（32.8%）：炎性假瘤8例，肺内淋巴结8例，结核病变2例，错构瘤2例，硬化性血管瘤1例。癌前病变18例（28.1%）。

## 3 讨论

### 3.1 肺部微小结节的诊断

肺部微小结节的良恶性鉴别是临床工作中的难点。仅仅从肺部微小结节患者的症状和体征上难以判断其性质，影像学检查如胸部CT是肺微小结节常规检查项目，通常根据结节的影像学特征如形状、大小、边缘、密度、有无钙化和空洞，结合患者年龄和病史判断其良恶性。良性结节CT上边缘光整整齐，清晰，有时可见长毛刺征; 恶性结节CT上可见毛玻璃状、分叶或切迹、短毛刺征、典型胸膜凹陷征及强化征等表现^[4]^。肿瘤标志物检测、痰细胞学检查及纤维支气管镜检查对外周肺微小结节的诊断价值欠佳。PET-CT在肺微小结节的诊治价值较CT高，但其较高的假阳性率，假阴性率和费用限制了其使用。据报道直径 < 5 mm结节仅1%为恶性，但5 mm-10 mm结节6%-28%为恶性^[5]^。本文统计病例中39.1%（25/64）为恶性肿瘤，28.1%（18/64）为癌前病变，32.8%（21/64）为良性病变，本文肺微小结节恶性病变较文献报道高，这可能和本中心对于肺微小结节行胸腔镜诊治的指征相关，对于肺部微小结节，本中心一般选取CT或PET-CT等影像学考虑恶性可能性大的肺微小结节行胸腔镜诊治，病理诊断是肺微小结节最后确诊的金标准，应用胸腔镜将肺部微小结节肺做楔形切除，既可以明确诊断，抓住治疗最佳时机，又能避免不恰当用药导致的药物副反应^[6]^。

### 3.2 肺微小结节的定位

应用胸腔镜诊治肺微小结节的关键在于如何准确定位和查找肺微小结节，尤其是结节位于肺实质深处时。回顾本文64例胸腔镜诊治肺微小结节经验，我们的体会是：①对于表浅、CT提示密度高的肺结节，术前根据胸部CT判断结节所在肺叶的解剖位置。进入胸腔探查时，肺完全萎陷时可观察到位于胸膜下的肿瘤稍突出于肺表面或有胸膜凹陷征，或伴有脏层胸膜色泽的改变，用卵圆钳在压缩的肺表面滑动或者轻夹病肺，实质性肿块不易压缩并有硬节感，或用低压力使肺膨胀后牵至切口附近用手指直接触摸病灶。②而对于距脏层胸膜深度 > 0.5 cm的小结节，或CT提示密度低的结节，常规于术前2 h用Hook-wire定位。对于一些密度较低的结节，发现即使将肺组织切下，仍难以找到结节，此时的关键是Hook-wire定位，且定位时Hook-wire务必贯穿肿块，组织切下时，可沿着穿刺针寻找结节，这样可以避免无法找到结节的尴尬。

### 3.3 胸腔镜手术治疗肺微小结节的优势

胸腔镜应用于临床多年，其在肺部疾病诊治的作用已被肯定，具有手术时间短、出血少、恢复快等优点，不增加住院费用和术中、术后并发症，是诊治肺微小结节的有效方法^[7]^，对于良性结节，电视辅助胸腔镜手术（video-assisted thoracic surgery, VATS）下肺结节楔形切除术在完整切除肿瘤的同时明确了诊断，达到诊断和治疗的双重目的，既消除了良性结节恶变的可能，解除了患者的思想顾虑，又最大限度保留了肺功能。对于恶性结节，胸腔镜可以明确诊断，继而进行及时、合理的治疗。目前VATS下肺癌根治性手术效果确切，5年生存率与传统开胸手术无明显差异，且并发症明显低于传统开胸手术^[8]^。一般而言，在同样达到肿瘤学根治的前提下，VATS手术相对于传统的开胸手术有独特的优势，采用小切口，不切断背阔肌、前锯肌等肌肉，不切断或撑开肋骨，损伤小，对于肩关节活动功能影响较小，同时因避免了肋骨断端的摩擦，患者术后疼痛较传统切口明显减轻，术后恢复快，住院时间短。术中肺楔形切除标本常规进行冰冻病理检查，根据病理诊断结果和患者全身情况做进一步处理。如为良性病变做肺的楔形切除术，若为原发性肺癌，患者肺功能能耐受肺叶切除，予其行胸腔镜下肺叶切除及淋巴结清扫; 若为原发性肺癌，但患者年龄大，肺功能较差，可予胸腔镜下肺楔形切除及淋巴结清扫术; 而若为转移性癌，予其胸腔镜下肺楔形切除术。肺微小结节的诊断和治疗可选择动态观察、活检、手术切除这三种方法^[9]^，对于直径≤0.5 cm的肺微小结节，由于其恶性可能性很小，可给予3个-6个月后复查，无变化则继续观察至1年。对于直径0.5 cm-1.0 cm的肺微小结节，根据影像学、病史等综合考虑低度危险的患者处理可类似于直径≤0.5 cm肺微小结节，而中、高危的患者（有肿瘤家族史、PET-CT提示高代谢、患者年龄大）经短期（2周左右）抗炎治疗，无效者可行3个-12个月间断复查，或可选择胸腔镜手术，以免延误治疗，对于其它部位恶性肿瘤术后的患者新出现的肺内微小结节，如肝癌术后、肠癌术后、乳腺癌术后新出现的肺内结节，考虑肺转移性瘤可能性大，若为单发或同侧的多发结节，考虑予其胸腔镜下楔形切除。总之，对于直径≤1.0 cm的肺部微小结节，特别是中、高危人群，在未经病理学确诊之前，恶性可能性大者; 短期观察是合理的，但应适当缩短观察期，尽量早期做出明确诊断及治疗^[10]^。外科手术切除仍然是可疑肺癌的外周型肺微小结节之首选诊断及治疗方法，以电视胸腔镜手术为代表的现代微创手术则能最大限度地消除患者的畏惧心理、减少手术创伤^[11]^。本文中64例患者均通过胸腔镜获得早期诊断和治疗，未出现死亡和严重并发症，因此胸腔镜对于高危肺微小结节的诊治具有重要价值，CT引到下Hook-wire定位可应用于距脏层胸膜深度 > 0.5 cm的小结节，或CT提示密度低的结节，以提高病变的切除率。

## References

[1] Munden RF, Pugatch RD, Liptay MJ (1997). Small pulmonary lesions detected at CT: clinical importance. Radiology.

[2] Tan BB, Flaherty KR, Kazerooni EA (2003). The solitary pulmonary nodule. Chest.

[3] Lv XY, Yang YH, H J (2011). Clinical application of CT-guided preoperative pulmonary nodule localization technique. Zhongguo Fei Ai Za Zhi.

[4] Chae EJ, Song JW, Seo JB (2008). Clinical utility of dual-energy CT in the evaluation of solitary pulmonary nodules: initial experience. Radiology.

[5] Wahidi MM, Govert JA, Goudar RK (2007). Evidence for the treatment of patients with pulmonary nodules: when is it lung cancer?. Chest.

[6] Yang DS, Li Y, Liu J (2010). Study on solitary pulmonary nodules correlation between diameter and clinical manifestation and pathological features. Zhongguo Fei Ai Za Zhi.

[7] Yang CK, Wu YL, Zhou HZ (2011). Video-assisted thoracoscopic surgery in the treatment of thoracic diseases: Clinical analysis of 94 cases. Shi Yong Yi Yuan Lin Chuang Za Zhi.

[8] Solaini L, Prusciano F, Bagioni P (2008). Video-assisted thoracic surgery of the lung: Analysis of intraoperative and postoperative complications over 15 years and review of the literature. Surg Endosc.

[9] Li Y, Shui XC, Yang DS (2010). Solitary pulmonary nodules: a risk factor analysis. Zhonghua Xiong Xin Xue Guan Wai Ke Za Zhi.

[10] Ginsberg RJ (2003). The solitary pulmonary nodule: can we afford to watch and wait?. J Thorac Cardiovas Surg.

[11] Varoli F, Vergani C, Caminiti R (2008). Management of solitary pulmonary nodule. Eur J Cardiothorac Surg.

